# Atypical Rash as a Presentation of Pityriasis Rosea in a 23-Month-Old

**DOI:** 10.7759/cureus.73526

**Published:** 2024-11-12

**Authors:** Kedar Tilak, Erica Ray, Majo Joseph

**Affiliations:** 1 Neonatology/Pediatric Infectious Diseases, Children's Mercy Kansas City, Kansas City, USA; 2 Pediatrics, The Brooklyn Hospital Center, New York, USA; 3 Pediatric Emergency Medicine, The Brooklyn Hospital Center, New York, USA

**Keywords:** atypical, benign, pediatric, pityriasis rosea, rash

## Abstract

Pityriasis rosea is a self-limiting skin disorder that can occur in pediatric patients. We report an atypical presentation of a 23-month-old male with a generalized rash similar in appearance to pityriasis rosea. We then review the literature on pityriasis rosea and its application to pediatrics. Our patient had the rash as the only presentation which was also atypical in appearance as opposed to the regular rashes seen with pityriasis rosea which makes this case further interesting. We only treated the case symptomatically and the patient had spontaneous resolution of symptoms.

## Introduction

Rashes are a common complaint for pediatric patients visiting the emergency department or outpatient clinic. Many rashes look similar, so promptly differentiating serious rashes from benign ones is vital. Pityriasis rosea (PR) is an acute, self-limiting disorder commonly described with an initial herald patch [1,2]. Although more often seen in the adult population, PR can occur in pediatric populations with a mean age of presentation of 10 years [2-4]. This case is a unique presentation of PR which was compiled after obtaining written consent from the parents. 

## Case presentation

We present a case of a 23-month-old otherwise healthy male patient who came to the emergency room (ER) with a one-week history of rash. The mother said this was the first time he had this kind of rash. She described the rash as red with raised lesions that initially started on the back and spread to the face and abdomen. The rash was non-pruritic, and the parents reported no associated symptoms. The parents denied fever, rhinorrhea, cough, vomiting, diarrhea, irritability, or lethargy.  

The child did not have any other medical history or history of surgery. He did not have any allergies to food or medication and had never been hospitalized. The parents reported that he did not take any medication regularly, which ruled out drug-related rashes. We also asked about exposure to allergens like plants, soaps, and detergents to rule out allergic reactions. The parents also had other children in the house,  and none had similar rashes or skin findings.  

**Figure 1:**
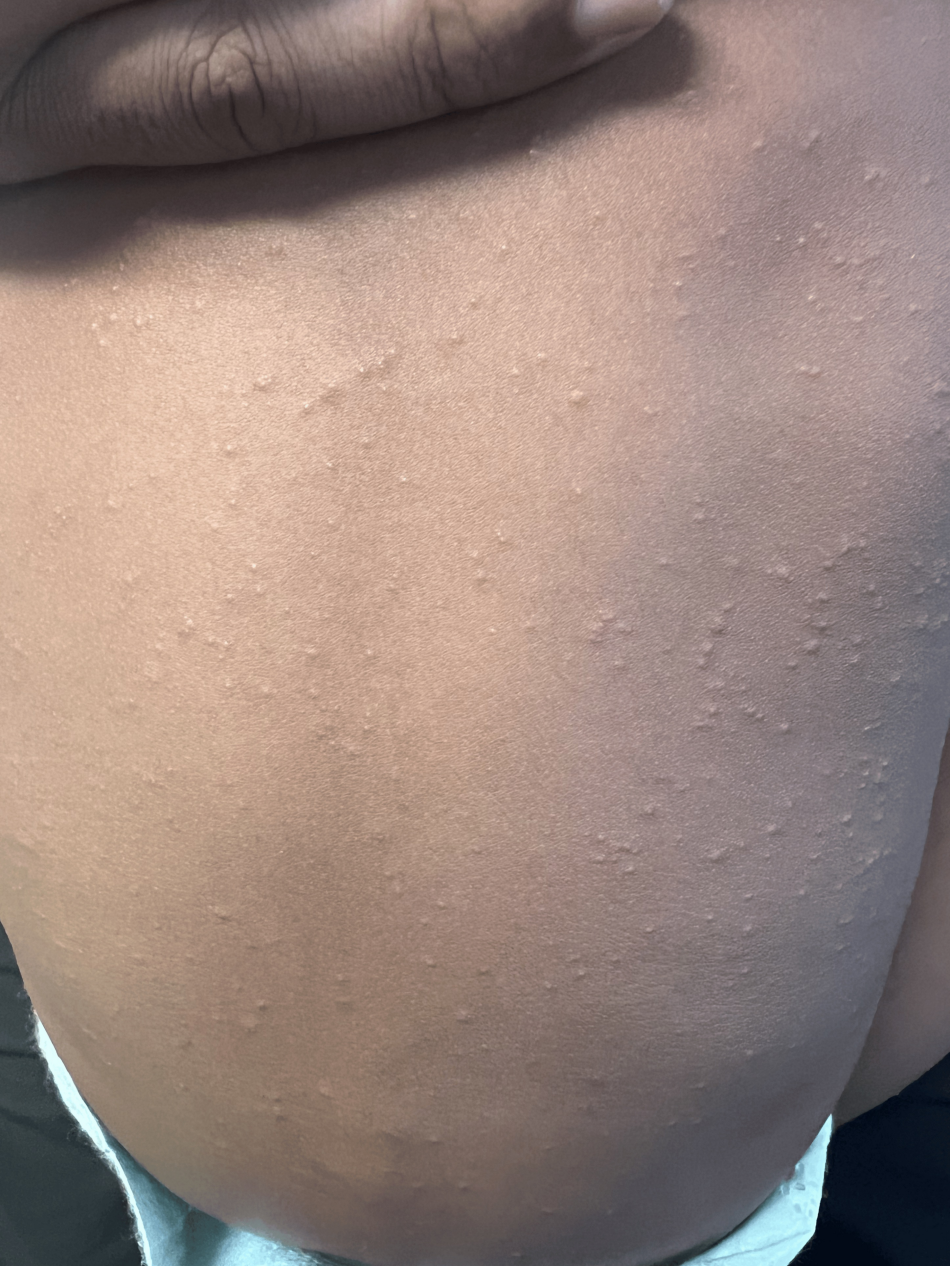
A papular, raised, non-erythematous, non-pruritic rash as seen over the back

**Figure 2:**
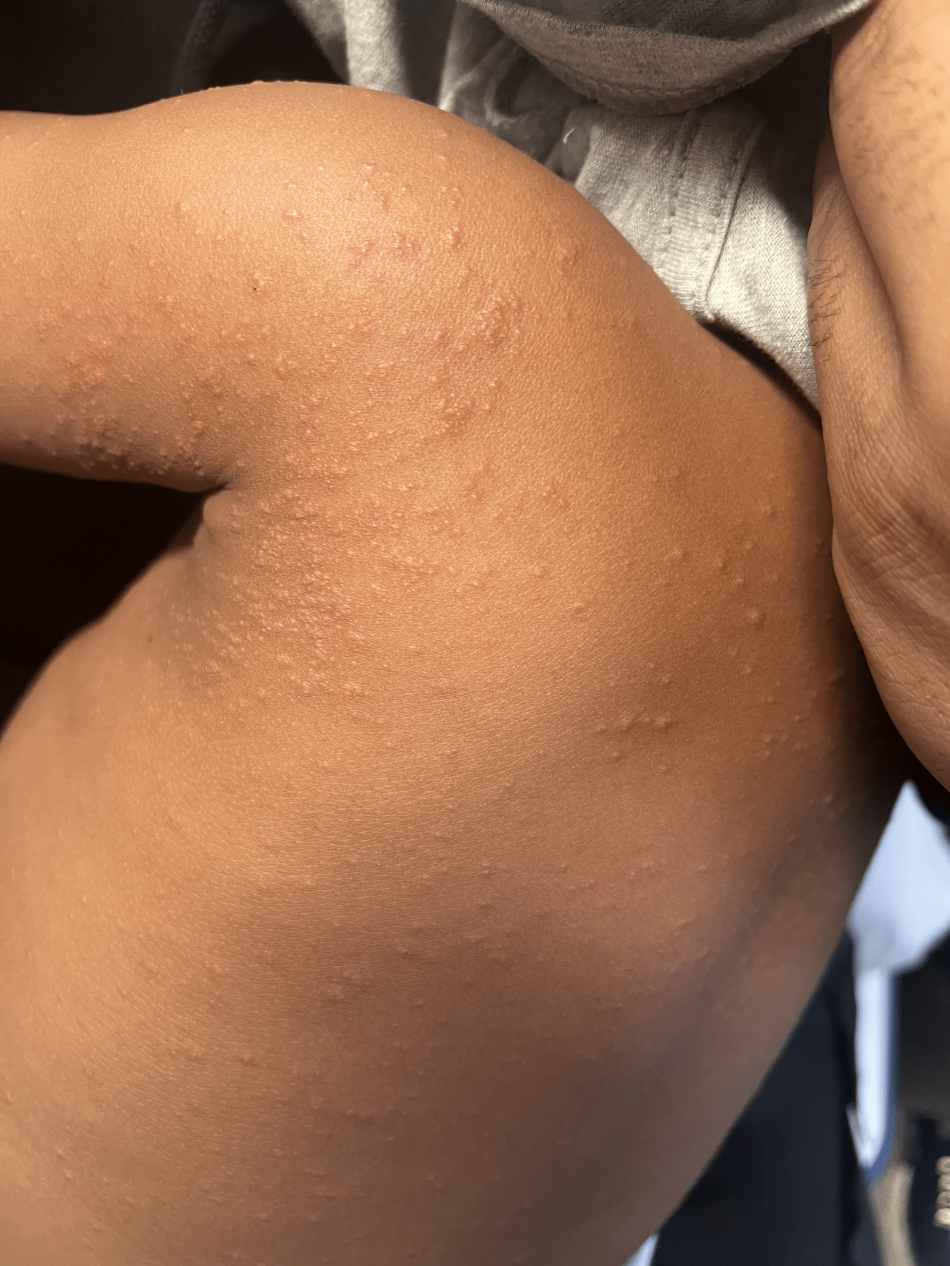
A papular, raised, non-erythematous, non-pruritic rash as seen over the left upper shoulder

**Figure 3:**
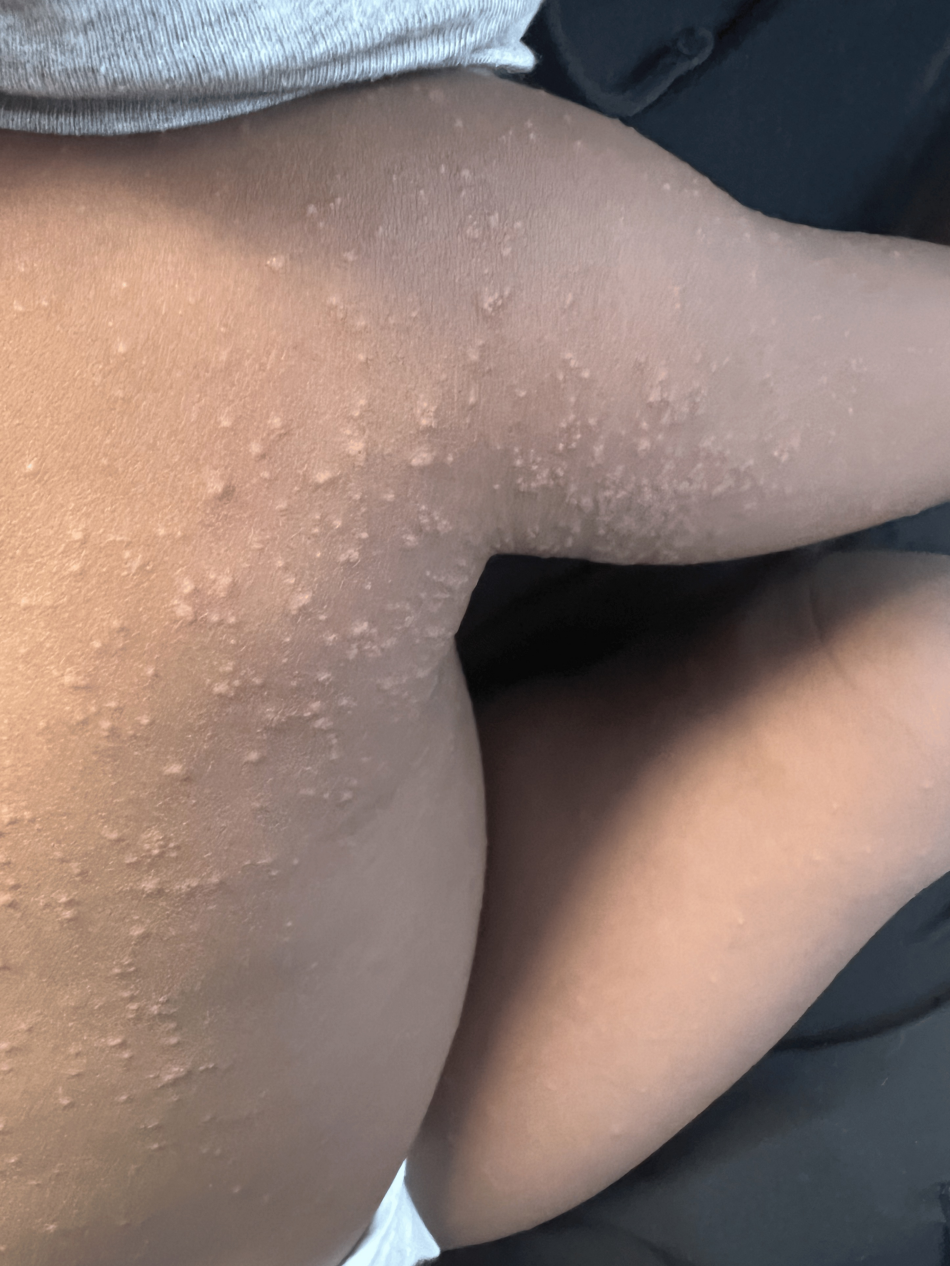
A papular, raised, non-erythematous, non-pruritic rash as seen over the right upper shoulder

On initial presentation to the ER, the patient’s vitals were within normal limits with a temperature of 37°C, heart rate of 103, and saturating oxygen at 100%. The patient appeared well and in no acute distress. He was playful and interactive. We performed a full review of the systems assessment and a comprehensive physical examination. His eyes did not have exudates; he had moist mucous membranes and no oral lesions, or rhinorrhea. He had equal chest rise, and the lungs were clear to auscultation bilaterally. He had no wheezes or murmurs and a regular heart rate and rhythm. The abdomen was soft, not tender, and not distended. He had no focal neuro deficits. Examination of the skin was remarkable for a nonspecific erythematous and papular rash on the face, back (Figure 1), upper left shoulder (Figure 2), upper right shoulder (Figure 3), and abdomen. There was no evidence of a herald patch. However, the mother reported the rash started in one area of the upper back and then progressively involved the rest of the areas. Otherwise, he was not scratching at the lesions. We did not notice any crusting or discharge from the rash. Also, there was no pathergy seen. The mother also said he did not have any birthmarks. The skin exam was also negative for Nikolsky’s sign and Darier’s sign.

We treated the patient symptomatically with a one-time dose of oral diphenhydramine (12.5 mg) and oral dexamethasone (8 mg) in the ER. Due to the child’s overall well-appearing nature and no additional symptoms, no laboratory testing was done. We sent a prescription of 12.5 mg diphenhydramine to be taken as needed every 6 h, in case of itching. The patient was advised to follow up with a pediatrician after three to five days. We also explained signs to look for and return precautions to the mother. We performed a thorough evaluation of the differential diagnosis for the rash. The diagnosis of PR was determined in the ER due to the rash having an initial lesion followed by the later development of the rest of the lesions and because the patient did not have any other associated symptoms like fever, congestion, or rhinorrhea.  

We contacted the mother to follow up on the rash after six weeks. The mother said the rash had eventually resolved, and the child had no residual skin findings. She also said that the child was healthy at baseline in the interim. 

## Discussion

Rashes are one of the most common complaints and reasons that prompt a visit to the ER, especially in the pediatric population. Clinicians must perform a full assessment of the rash, including the location of the rash, clinical presentation, any other associated signs and symptoms, and any exposures [5]. One of the most important considerations for any rash, especially in the pediatric population, is determining if the patient needs an urgent evaluation. Certain rashes, like allergies, present with hives and other associated symptoms like vomiting. Additionally, shortness of breath or mucosal changes might be associated with more serious conditions like Steven-Johnson syndrome [6]. PR mainly affects children and young adults aged 10 to 35 years, with the highest incidence in adolescents (10-19 years) and is less common in children under 10 years. Seasonal peaks are noted in spring and fall, with geographical variability. The rash typically begins with a herald patch, a single oval, pink or salmon-colored lesion (2-10 cm) with a scaly border, followed by smaller lesions appearing within one to two weeks. The rash often has a “Christmas tree” pattern on the trunk and usually spares the face and scalp. Lesions are raised, well-defined, and may cause mild itching, but the condition is generally asymptomatic and self-limiting, resolving in six to 12 weeks.

When our patient presented to the ER, the rash was his only complaint. The first thing we observed was the clinical appearance of the rash. We did not see any associated urticaria or itching and we ruled out allergic causes [5]. He did not have any fever or upper respiratory symptoms. The rash was not macular and had no specific pattern of spread, which ruled out roseola and most other viral causes [7]. There was no mucosal involvement, and the child was very well-appearing and had no recent drug exposures, which ruled out serious causes like Steven-Johnson syndrome, toxic epidermal necrolysis, and other drug-associated rashes [6].  

As mentioned before, PR usually presents as an initial patch followed by the development of bilateral, symmetric lesions on the back along the Langer lines [5]. Classically, the herald patch is described as an erythematous circular lesion with slightly raised and scaly edges, most notably found on the upper back or trunk. This initial lesion can then spread into a “Christmas tree” pattern to the rest of the trunk and proximal extremities [1]. However, it is important to note that the herald patch is not always present in PR due to the various classifications of the disorder [8]. Additionally, the rash can be associated with a prodrome of symptoms, including fever, malaise, and sore throat in some cases [9]. 

Although linked with viral etiologies, such as human herpesvirus 6 (HHV 6), PR can also occur secondary to certain medications and vaccinations [9,10]. In addition to etiology, PR can also be subdivided into distinct categories dependent on the presentation and relapsing or persistent nature of the presentation [10]. These categories include classic, relapsing, persistent, pediatric, pregnancy-related, and PR-like eruption [10]. The pediatric type of PR is commonly associated with a recent infection and eruption of the rash on the trunk and limbs, with approximately 60% of cases starting with an initial herald patch and half of the cases associated with systemic symptoms [10].   

Our case was atypical due to the patient’s young age and the scant number of associated symptoms. As previously stated, the mean age for PR in pediatric patients is approximately 10 years [3,4]. In a 23-month-old child, differential diagnoses for rashes could include atopic dermatitis, contact dermatitis, and psoriasis. The rash that was seen in our patient was not scaly which can be seen in seborrheic dermatitis. Also, lichen planus is usually pruritic and purplish, molluscum contagiosum has a central umbilication, and dermatofibromas are small brownish and firm. In addition to these classical rashes, pediatric rashes can also be subdivided into pathogenic causes, such as viral, bacterial, or fungal rashes [5,7]. In addition to serving as an uncommon diagnosis in our patient, the distribution on the face was also an atypical presentation compared to the typical limb and trunk involvement [10]. However, in a study conducted by Amer et al., which looked at PR in 50 African American children, approximately 30% of their patients had rashes with facial involvement, 33% had papular lesions, and 48% had residual hyperpigmentation [11]. 

As was the case in our patient, the typical course of PR is self-limiting and usually involves watchful waiting for the resolution of symptoms [9]. The average course for pediatric PR is 16 days, with some cases lasting up to six to eight weeks [9,10]. Overall, the initial herald patch, lack of systemic symptoms, and self-resolution of the rash in our 23-month-old patient made PR a favorable diagnosis.

## Conclusions

In summary, PR is a benign, self-limiting disorder of the skin most often found in the adult population. Although less common in the pediatric population, it remains important for clinicians to understand the disorder and be able to identify it in younger patients. As mentioned, the case we presented is atypical due to a few factors but provides a learning opportunity regarding the various etiologies of pediatric rashes. This remains an important topic to discuss as it provides clinicians with the tools to differentiate between benign and serious rashes. 
